# Exploring the combined anti-cancer effects of sodium butyrate and celastrol in glioblastoma cell lines: a novel therapeutic approach

**DOI:** 10.1007/s12032-024-02340-6

**Published:** 2024-03-26

**Authors:** Bahar Kartal, Farika Nur Denizler-Ebiri, Mustafa Güven, Filiz Taşpınar, Hande Canpınar, Sedat Çetin, Tuğçe Karaduman, Serkan Küççüktürk, Javier Castresana, Mehmet Taşpınar

**Affiliations:** 1 Department of Medical Biology, Faculty of Medicine, Yuzuncu Yıl University, Van, Turkey; 2 Faculty of Medicine, Yuzuncu Yıl University, Van, Turkey; 3 Department of Physiology, Faculty of Medicine, Aksaray University, Aksaray, Turkey; 4 Department of Basic Oncology, Faculty of Medicine, Hacettepe University, Ankara, Turkey; 5 Department of Biochemistry, Faculty of Veterinary Medicine, Yuzuncu Yıl University, Van, Turkey; 6 Molecular Biology and Genetics, Faculty of Sciences and Letter, Aksaray University, Aksaray, Turkey; 7 Department of Medical Biology, Faculty of Medicine, Karamanoğlu Mehmetbey University, Karaman, Turkey; 8 Department of Biochemistry and Genetics, University of Navarra, Pamplona, Spain; 9 Department of Medical Biology, Faculty of Medicine, Aksaray University, Aksaray, Turkey

**Keywords:** Glioblastoma, Sodium butyrate, Celastrol, DNA repair, Apoptosis, Autophagy

## Abstract

Glioblastoma, a highly aggressive and lethal brain cancer, lacks effective treatment options and has a poor prognosis. In our study, we explored the potential anti-cancer effects of sodium butyrate (SB) and celastrol (CEL) in two glioblastoma cell lines. SB, a histone deacetylase inhibitor, and CEL, derived from the tripterygium wilfordii plant, act as mTOR and proteasome inhibitors. Both can cross the blood–brain barrier, and they exhibit chemo- and radiosensitive properties in various cancer models. GB cell lines LN-405 and T98G were treated with SB and CEL. Cell viability was assessed by MTT assay and IC50 values were obtained. Gene expression of DNA repair, apoptosis, and autophagy-related genes was analyzed by RT-PCR. Cell cycle distribution was determined using flow cytometry. Viability assays using MTT assay revealed IC50 values of 26 mM and 22.7 mM for SB and 6.77 μM, and 9.11 μM for CEL in LN-405 and T98G cells, respectively. Furthermore, we examined the expression levels of DNA repair genes (MGMT, MLH-1, MSH-2, MSH-6), apoptosis genes (caspase-3, caspase-8, caspase-9), and an autophagy gene (ATG-6) using real-time polymerase chain reaction. Additionally, flow cytometry analysis revealed alterations in cell cycle distribution following treatment with SB, CEL and their combination. These findings indicate that SB and CEL may act through multiple mechanisms, including DNA repair inhibition, apoptosis induction, and autophagy modulation, to exert their anti-cancer effects in glioblastoma cells. This is the first study providing novel insights into the potential therapeutic effects of SB and CEL in glioblastoma.

## Introduction

Glioblastoma (GB) is the most common and lethal primary brain cancer [1]. GB is classified as grade IV cancer based on World Health Organization guidelines [2] and shows high recurrence rates and resistance to a spectrum of therapeutic modalities. The current treatment strategy of GB is based on utilizing concurrent chemotherapy and radiotherapy after maximal surgical resection followed by adjuvant temozolomide therapy [3]. With the advancements in GB treatment, median survival increased in the last two decades. Nevertheless, the overall prognosis remains poor. Furthermore, studies targeting GB with novel therapies to improve prognosis have failed [4]. Hence, new treatment options and strategies are needed in the way to find a cure for GB.

An increasing number of studies have demonstrated the efficacy of targeting histone deacetylases (HDAC) via HDAC inhibitors (HDACi) [5]. Chemo- and radiosensitive properties of HDACi can be exploited for better cancer treatments for GB. Sodium butyrate (SB), an HDACi derived from a fatty acid, has gained attention due to its ability to inhibit class I and IIa HDACs [6, 7]. Preclinical investigations have established the anti-neoplastic effects of sodium butyrate, both as a monotherapy and in combination with other chemotherapeutic agents, across various cancer types [8–12]. While the blood–brain barrier (BBB) represents a critical impediment to achieving effective treatments for GB [4], SB effectively overcomes this hurdle by exhibiting a remarkable ability to readily cross the BBB [13]. Although several effects of SB have been elucidated in preclinical cancer models, further investigations are warranted to validate the therapeutic potential of SB in the management of GB.

Celastrol (CEL), Chinese herbal medicine, is a triterpenoid and the most promising bioactive component isolated from the tripterygium wilfordii plant. CEL is an mTOR inhibitor and a proteasome inhibitor [14, 15] and has potent anti-inflammatory effects. CEL has been used in obesity, several inflammatory diseases like Crohn ‘s disease, rheumatoid arthritis, and many other diseases with preventive and therapeutic purposes [16]. Recently, a growing body of literature also recognizes the anti-cancer effects of CEL on various cancers [17]. We have previously shown that CEL alone and in combination with 5-Floururacil has decreased viability in GB cells [18]. CEL inhibits angiogenesis, vasculogenic mimicry, and tumor growth in GB models [19]. Further studies are needed to elucidate molecular mechanisms and therapeutic effects of CEL in GB.

The combined use of SB, HDACi, and CEL as a proteasome inhibitor could serve as a significant model in the development of cancer treatment approaches. This is because the potential activation of tumor suppressor genes by SB and the accumulation of ubiquitinated proteins through proteasomal inhibition by CEL may present a novel therapeutic modality for GB treatment. Therefore, this study aims to investigate the likely therapeutic effect of the combined use of SB and CEL on GB cells. Herein, we have shown for the first time the anti-cancer effects of SB and CEL via the investigation of DNA repair, apoptotic, autophagic, and cell cycle effects in GB cell lines.

## Materials and methods

### Cell culture and reagents

LN-405 and T98G glioblastoma cell lines were used in this study. In an incubator with 5% CO2 at 37 °C, 95% humidity environment, in addition to 10% FBS, 1% Penicillin–Streptomycin, LN-405, and T98G cell lines were grown in DMEM media, and in RPMI-1640 media respectively.

SB (B5887) and CEL (C0869) were purchased from Sigma-Aldrich. SB and CEL were prepared by dissolving in water and DMSO, respectively, according to the manufacturer ‘s protocol. 5, 10, 15, 20, 25, 30, 40, and 50 mM for SB; 0.5, 1, 2, 3, 4, 5, 6, 8, and 10 µM concentrations were used for CEL.

### Viability assay with MTT

MTT (3-(4,5-dimethylthiazol-2-yl)-2,5-diphenyltetrazolium bromide) analysis was performed, and inhibitory doses IC_50_ (Inhibitory dose) of SB and CEL were determined on LN-405 and T98G cell lines. Trypsinized cells were seeded in 96-well plates with 8000 cells per well for MTT analysis. After overnight incubation at 37 °C, the cells in the plates were serum starved for 8 h to equalize the cell cycles. At the end of this period, 5, 10, 15, 20, 25, 30, 40, and 50 µM for SB; 0.5, 1, 2, 3, 4, 5, 6, 8, and 10 µM concentrations were prepared for CEL. The prepared concentrations were given to the cells in 100 µl of medium in each well of the plate. At the end of the 72-h incubation period, the medium was discarded, and the MTT mixture was added to each well with a final MTT concentration of 0.5 mg/ml in 100 µl of medium, and the cells were incubated at 37 °C for 3 h in the incubator. At the end of the period, the medium was discarded, and 100 µl of lysing solution was added to each well. Thus, formazan crystals formed by living cells were dissolved. After the formazan crystals were dissolved, the absorbance values of each well at 570 nm were determined in the spectrophotometer. The IC_50_ values of the cells were determined from the absorbance values with the GraphPad Prism program.

### Total RNA isolation and cDNA synthesis

SB and CEL molecules alone and in combination at concentrations of IC50 values were applied to T98G and LN-405 cell lines and incubated for 72 h. Trizol was used for total RNA isolation, and qualitative evaluation was performed using 1% agarose gel. The quantity and purity of RNAs were evaluated using the Biodrop device. To determine the expression levels of targeted genes, cDNA synthesis was performed with the GeneAll HyperScriptFirst Strand Synthesis Kit (Catalog: 601–005) according to the manufacturer ‘s protocols.

### Real-time polymerase chain reaction (RT-PCR)

Gene expression analyses were performed in LN-405 and T98G cell lines after 72 h of incubation at IC_50_ values of SB and CEL alone or in combination. DNA repair (MLH1, MSH2, MSH6, and MGMT), apoptosis (caspase 3, caspase 8, and caspase 9), and autophagy (Atg6) pathway gene expressions were examined in both cell lines. GAPDH was determined as the control gene. Gene RealAmp SYBR qPCR Master mix kit was used to determine gene expression patterns. Three independent replicates were made for each sample. The 2-ΔΔCt formulation was used to determine the relative difference that may occur between the expressions.

### Flow cytometry

The cells were seeded in 25 cm^2^ flasks at a density of 1X106 cells and treated with SB and CEL at IC50 concentrations. The cells were then incubated for 72 h. At the end of the 72-h incubation period, the cells were trypsinized and centrifuged at 1500 rpm for 5 min, and the supernatant was discarded. The cell pellet was resuspended in 3 ml of sterile PBS (without Ca+2 and Mg+2) and centrifuged again at 1500 rpm for 5 min. This process was repeated twice. Afterward, the supernatant was discarded, and 2 ml of sterile PBS (without Ca+2 and Mg+2) was added to the pellet. The supernatant was discarded again, and the pellet was resuspended by pipetting after adding 70 μl of RNase and 50 μl of Propidium Iodide. Subsequently, the samples were incubated at room temperature in the dark for 20 min. After incubation, the cells were filtered through a 37 μm nylon mesh and transferred to a flow cytometer for analysis. Lastly, 10,000 cells were counted on a flow cytometry device (EPICS XL MCL, Beckman Coulter), and cell cycle and DNA analysis were performed. Bivariate DNA histograms of the ratio of cells in G0/G1, synthesis, and G2/M phase and the ratio of apoptotic cells were analyzed using the MCYCLE program (Phonex Sys).

### Statistics

The SPSS 15.0 package program was employed to conduct the analysis of the obtained results. To examine the distribution of categorical variables among different groups, either the Chi-square test or Fisher ‘s exact test was employed. The outcomes are presented in tabular form, with descriptive statistics such as frequency distributions and percentages. Group comparisons for each genotype were performed using one-way ANOVA. Tukey ‘s post-hoc test was used for pairwise comparisons. A significance level of *p* < 0.05 was deemed statistically significant for this study.

## Results

### Cytotoxic effects of sodium butyrate and celastrol

**Fig. 1:**
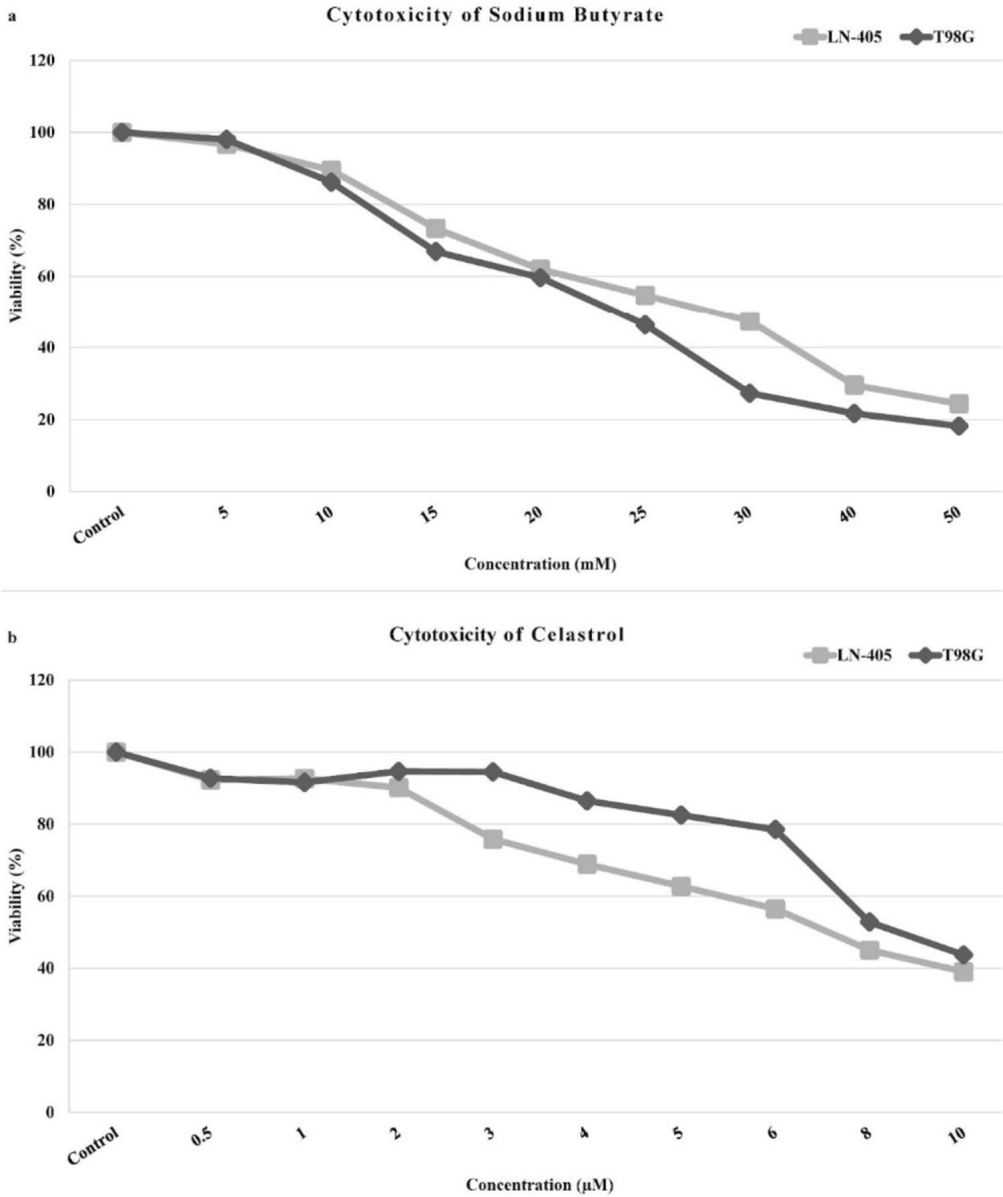
Cytotoxicity of SB and CEL in LN-405 and T98G cell lines. MTT experiments were used to determine the cytotoxicity of SB (**a**) and CEL (**b**). Absorbance was measured at 72 h

The cytotoxic effect of SB and CEL was detected in both cell lines at 72 h of incubation following drug administration. The IC_50_ values of SB and CEL were found to be 26 mM and 6.77 μM and 22.7 mM and 9.11 μM for LN-405 and T98G cell lines, respectively (Fig. 1).

## Gene expressions

### DNA repair genes (MGMT, MLH-1, MSH-2, MSH-6)

**Fig. 2:**
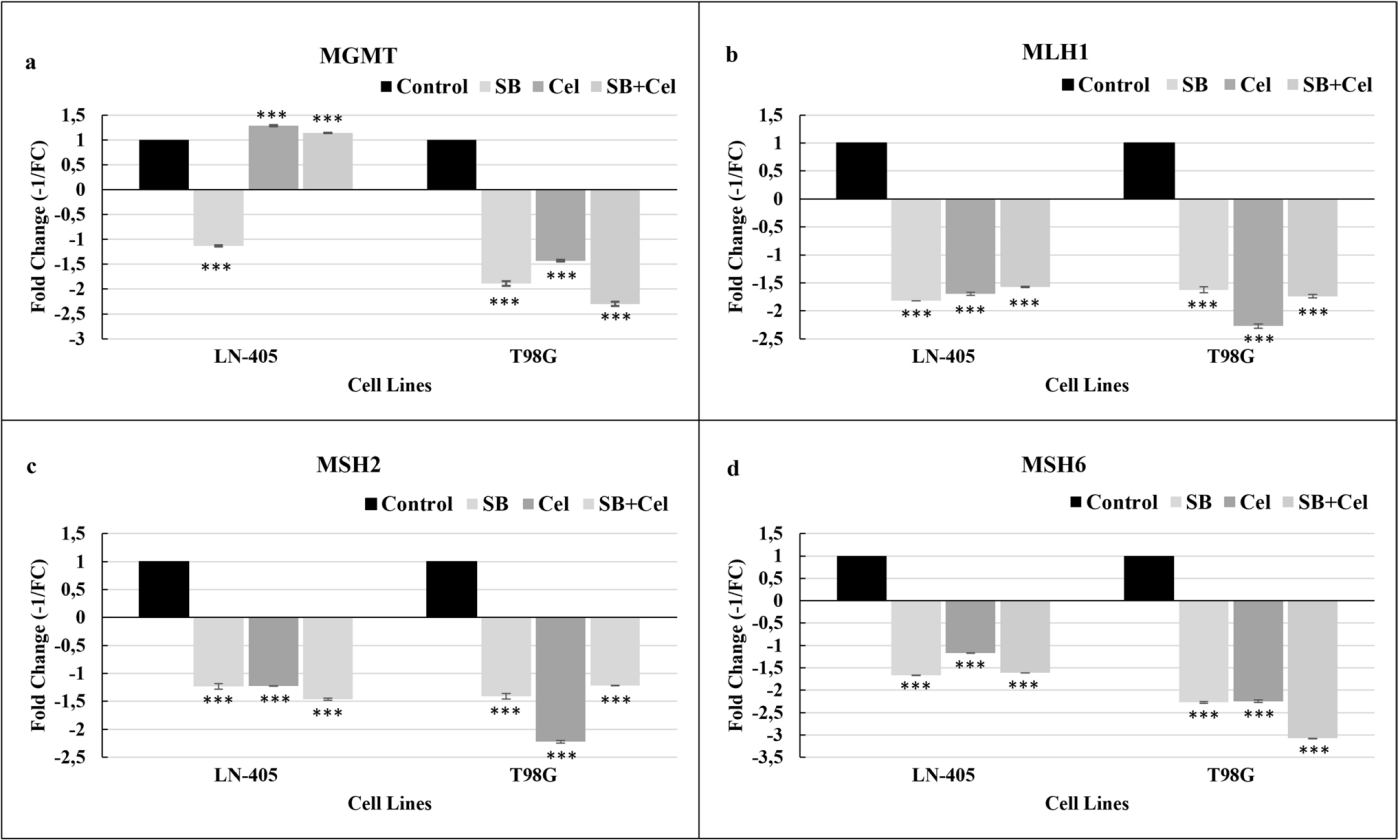
DNA repair gene expression levels in groups treated with SB, CEL, and SB+CEL combination in LN-405 and T98G cell lines. The cells were treated with IC50 values of SB and CEL. (**a**) MGMT, (**b**) MLH-1, (**c**) MSH-2, (**d**) MSH-6. Significance is shown only for relationships between the control group and other groups. *, **, and *** denotes *p* < 0.05, *p* < 0.01, and *p* < 0,001 respectively

In both the LN-405 and T98G cell lines, a significant difference was observed in terms of MGMT gene expression between the control and SB groups, control and CEL groups, and control and SB+CEL groups (*p* < 0.001). Notably, all pairwise comparisons yielded significant results (*p* < 0.001) (Fig. 2a).

Furthermore, in the T98G cell line, a significant difference was found in terms of MGMT gene expression between CEL and SB groups, as well as between CEL and SB+CEL groups (*p* < 0.005 and *p* < 0.001, respectively).

Significant differences were observed in MLH-1 expression between the control group and all other groups in the LN-405 and T98G cell lines (*p* < 0.001). Moreover, a significant relationship was detected between SB+CEL and both SB and CEL groups in the LN-405 cell line (*p* < 0.001 and *p* = 0.038, respectively) (Fig. 2b). In the T98G cell line, a significant association was found in terms of MLH-1 expression between the CEL group and both SB and SB+CEL groups (*p* = 0.006, *p* = 0.023), respectively.

In both the LN-405 and T98G cell lines, there were notable and statistically significant variations observed in MSH-2 expression between the control group and all other groups (*p* < 0.001) (Fig. 2c). Moreover, significant relationships were detected between SB+CEL and both SB and CEL in both the LN-405 and T98G cell lines regarding MSH-2 expression (*p* = 0.007 and *p* = 0.005 for LN-405; *p* = 0.016 and *p* < 0.001 for T98G cells).

In both the LN-405 and T98G cell lines, significant and pronounced differences were observed in MSH-6 expression between the control group and all other groups (*p* < 0.001) (Fig. 2d). Similarly, a significant relationship was detected between SB+CEL and both SB, as well as between SB+CEL and CEL, in both cell lines (*p* < 0.05).

### Apoptosis genes (CASP-3, CASP-8, CASP-9)

**Fig. 3:**
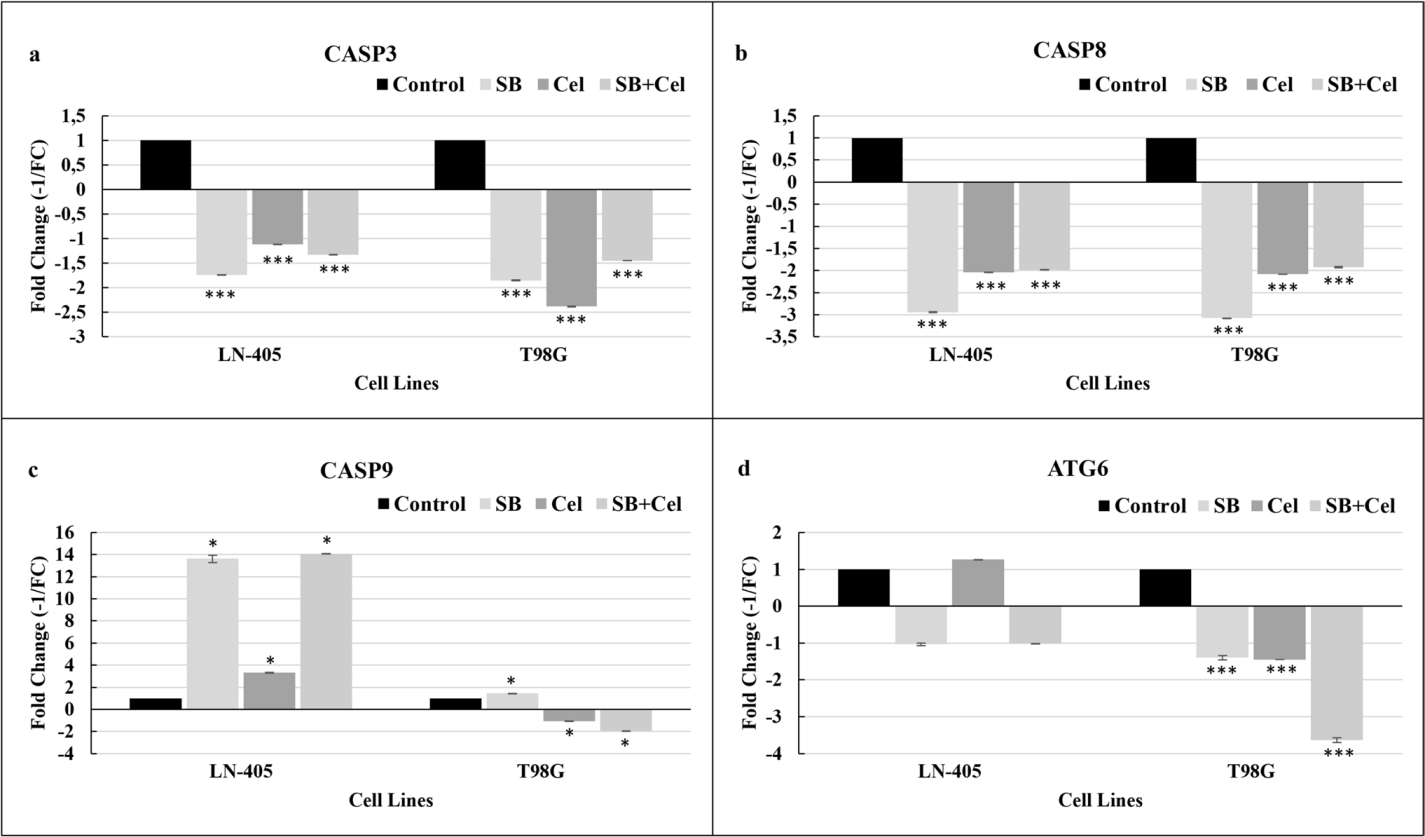
Apoptosis and autophagy gene expression levels in groups treated with SB, CEL, and SB+CEL combination in LN-405 and T98G cell lines. (**a**) CASP-3, (**b**) CASP-8, (**c**) CASP-9, and (**d**) ATG-6. Significance is shown only for relationships between the control group and other groups. *, **, and *** denotes *p* < 0.05, *p* < 0.01, and *p* < 0,001 respectively

A statistically significant difference was observed in CASP-3 gene expression between the control group and all other groups in both the LN-405 and T98G cell lines (*p* < 0.001) (Fig. 3a).

Significant differences were noted in CASP-8 gene expression between the control group and all other groups across the LN-405 and T98G cell lines (*p* < 0.001) (Fig. 3b). Furthermore, a significant relationship was observed between SB and CEL, as well as between SB and SB + CEL for both cell lines (*p* < 0.001).

Additionally, a significant relationship was detected between CEL and SB + CEL only in the T98G cell line (*p* = 0.007).

In both the LN-405 and T98G cell lines, a statistically significant difference was observed in CASP-9 gene expression between the control group and all other groups (*p* < 0.05) (Fig. 3c). Furthermore, a consistent and significant relationship was detected between CEL and SB, as well as between CEL and SB+CEL, across both cell lines (*p* < 0.001 for all pairwise comparisons).

### Autophagy gene (ATG-6)

In the LN-405 cell line, a statistically significant relationship was observed between CEL and all groups (*p* < 0.001). In the T98G cell line, this relationship was found to be statistically significant between the control and all groups, as well as between SB and SB+CEL and between SB and SB+CEL combinations (*p* < 0.001) (Fig. 3d).

### Cell cycle analysis

Cells were treated with SB and CEL at their respective IC50 values in both cell series, and cell cycle analysis was conducted using flow cytometry (Table 1). In the LN-405 cell series, significant differences in cell cycle distribution were observed between SB and CEL (*p* = 0.015), as well as between the control and SB (*p* = 0.006), and the control and SB+CEL (*p* = 0.014) groups (Table 2). In the T98G cell series, significant differences in cell cycle distribution were found between the control and CEL (*p* = 0.012), the control and SB+CEL (*p* = 0.001), SB and SB+CEL (*p* = 0.028), and CEL and SB+CEL (*p* = 0.042) groups (Table 2).

**Table 1:**
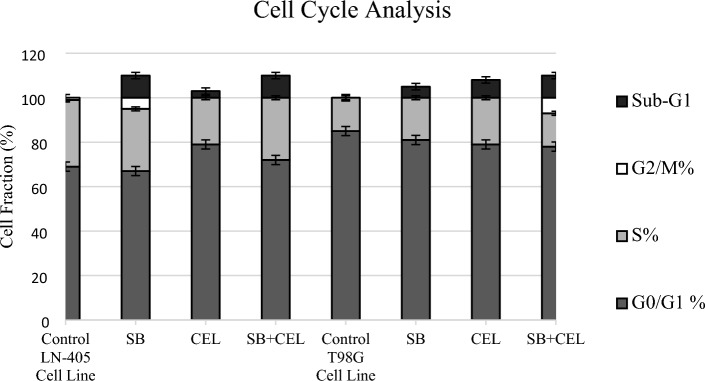
Cell fraction is expressed as the percentage of the cells. The cells were treated with IC50 values of SB and CEL on LN-405 and T98G cell lines

**Table 2:** p values of cell cycle analysis in LN-405 and T98G cell lines following SB, CEL, and SB+CEL treatments

Groups	Pairwise Comparisons	LN-405	T98G
Control	SB	0.006	0.066
	CEL	0.101	0.012
	SB+CEL	0.014	0.001
SB	CEL	0.015	0.679
	SB+CEL	0.159	0.028
CEL		0.088	0.042

## Discussion

GB is a highly aggressive brain tumor with limited therapeutic options and a poor prognosis. Although different approaches have been developed in the recent years, GB patients have a low overall survival rate. Approaches modifying epigenetics have been gaining importance. Targeting HDACs is one of the most significant approaches. SB is an HDAC inhibitor that has demonstrated anti-neoplastic effects in various cancers, including GB. CEL, on the other hand, is an mTOR and proteasome inhibitor with anti-inflammatory and anti-neoplastic properties. In our study aiming to propose a different approach for GB treatment, we aimed to assess the combined effects of SB and CEL, which have not been previously investigated in any cancer type.

In our study, the IC50 value of SB was found to be 26 mM for the LN-405 cell line and 22.7 mM for the T98G cell line. Previous studies have reported that the IC50 value of SB varies depending on time and dose, with a range of 1.5–20 mM [8, 9, 20–22]. In our study, it was observed that T98G cells were more sensitive to SB compared to LN-405 cells. Although both of these cells are of GB origin, the difference in SB sensitivity indicates that their genomic and epigenomic structures are different, and these differences can lead to changes in many steps, from signaling pathways to proliferation capabilities, and therefore their responses to drugs may also differ. In our study, the IC50 value obtained for SB at 72 h was found to be higher compared to the literature. This discrepancy is presumed to be due to the genetic and epigenetic structures of LN-405 and T98G cells, as well as the diversity of the test method and other cell culture factors used in the study.

Studies have demonstrated that CEL exhibits cytotoxicity in different cancer cell lines, with varying IC50 values, ranging from 1.2 to 5.6, depending on the duration of exposure [23–28]. In the present study, distinct responses to CEL were observed in two different cell lines, with IC50 values of 6.77 μM for LN-405 and 9.11 μM for T98G. These findings suggest that the response to CEL may be influenced by metabolic and genomic heterogeneity, indicating the potential influence of variations in cellular characteristics on drug sensitivity. Importantly, the obtained IC50 values of CEL in this study were consistent with those reported in the literature, strengthening the reliability and validity of the findings.

To the best of our knowledge, there are no studies in the literature investigating the effects of SB in the context of MGMT, MLH1, MSH2, and MSH6 gene expressions. However, one study indirectly attempted to explain the anti-apoptotic mechanism of SB on glioma [29]. It was suggested that SB induces the methylation of the HEY1 gene, leading to increased DNA methyltransferase expression and subsequent anti-apoptotic effects through the methylation of oncogenes. Furthermore, the methylation of the HEY1 gene, which increases the expression of HDACs, has been reported to result in the decreased expression of HDACs and thus indirectly contribute to the HDAC inhibitory and anti-apoptotic effects of SB [29]. Studies conducted on colon cancer have presented conflicting results regarding the relationship between SB and repair genes [30, 31]. Coxhead et al. (2005) reported that SB is more effective in MMR-negative cells, while Sun et al. (2018) demonstrated that SB did not induce apoptosis in MLH1-deficient cells [30, 31]. Bultman and Jobin (2014) proposed that microbial-derived SB triggers the proliferation of epithelial cells in the colon, suggesting that SB may be an oncometabolite [32]. Studies on colon cancer have shown that HDAC inhibition via SB can occur through methylation [31] and that SB also has the potential to act as a methylation agent [33]. The data proposed regarding the cellular effects of SB further emphasizes the importance of investigating the effect of SB on GB. In our study, SB treatment reduced the expression of MGMT, MLH1, MSH2, and MSH6 genes in both cell lines. The decreased expression of repair genes may be associated with the methylation potential proposed for SB in colorectal cancer. Similarly to SB, there is a lack of studies in the literature investigating the effects of CEL on the expression of MGMT, MLH1, MSH2, and MSH6 genes. After CEL treatment, the expression of the MGMT gene was similar to the control in both cell lines. CEL reduced the expression of MLH1, MSH2, and MSH6 genes in both cell lines. This result suggests that CEL can be used in combination with radiotherapy for GB treatment. When SB and CEL were used together, the expression of the MGMT gene was the same as the control in LN-405 cells but decreased in T98G cells. This result indicates that the combination of SB and CEL can overcome MGMT-mediated resistance in GB. However, this combination partially reduced the expression of MLH1, MSH2, and MSH6 genes. In general, the decrease in MMR gene expression in cancer cells can lead to the accumulation of new mutations. These new mutations can either lead to cell death or increase genomic instability, making the cell more susceptible to radiotherapy. Therefore, SB and CEL can be used together in GB. With this in hand, further studies are needed to better elucidate the relationship between SB and CEL. Additionally, further investigations, including the base excision repair and PARP genes in the pathway, are required for a more detailed understanding.

It is known that HDAC activity is increased in cancer cells [10]. Therefore, using an HDAC inhibitor in cancer treatment is a rational therapeutic approach. Although the therapeutic relationship of SB as an HDAC inhibitor is known in the literature, there are limited studies regarding its apoptotic effects in GB [10, 29, 34]. It has been indicated that SB inhibits cell proliferation and induces senescence in the A172 glioma cell line, suggesting that the use of SB in GB could be a treatment strategy [11]. In this study, it was found that after SB was applied in both cell lines, cells entered the apoptotic process via Caspase 9 without autophagy. It is expected that a methyltransferase and HDAC inhibitor agent like SB induces apoptosis without autophagy, which is consistent with the literature [10, 29, 34].

There are several studies available on the mechanism of cell death in cancer cells with CEL. Some of these publications report that CEL induces apoptotic cell death [17, 35–38], while others indicate its induction of autophagy [39–41]. Additionally, it has been noted that CEL induces the precursor death model of paraptosis before apoptosis and autophagy [42]. Studies have also been conducted on CEL in GB samples, demonstrating its induction of apoptosis (Cha et al., 2019) and autophagy [43]. In this study, it was observed that the expression of Caspase 3 in LN-405 cells treated with CEL was at the same level as the control group, while the expression of Caspase 9 increased threefold compared to the control. In T98G cells, no change in Caspase expression was detected. The lack of change in Caspase expression suggests that CEL may exert cell-specific effects. CEL treatment was shown to induce autophagy and apoptosis in HeLa cells parallel to paraptosis [42]. These processes are likely due to the mechanism of action of proteasome inhibitor molecules such as CEL. Accumulation of ubiquitinated proteins leads to endoplasmic reticulum-mediated vacuolization, and autophagy becomes an inevitable choice for cells that want to rescue their organelles. Prolonged autophagy will also trigger apoptosis.

In a limited number of studies demonstrating the combination use of HDAC inhibitors (HDACi) with proteasome inhibitors in GB treatment [44–46], it has been reported that the co-administration of the proteasome inhibitor Bortezomib with HDACi molecules increases DNA damage and induces apoptosis in glioma cells. A recent report suggests that in silico analysis of the combined use of HDACi and proteasome inhibitors could be effective in p53 mutant GB treatment [47]. According to the limited literature available, the combination of HDACi and proteasome inhibitors represents an important alternative treatment strategy for GB. Therefore, in our study, we selected SB, an HDACi, and CEL, a potent proteasome inhibitor, which both can cross BBB easily. When SB and CEL were used together in LN-405 cells, it was observed that the apoptotic effect of SB continued in the presence of CEL, but in T98G cells, it decreased compared to the control. This indicates that T98G cells are more resistant to both CEL and SB when combined, compared to LN-405 cells. This finding is also supported by cytotoxicity studies. The decrease in Atg6 expression upon the combination use of SB and CEL suggests that SB may reduce the potential autophagic effect of CEL. The results of both apoptosis and autophagy indicate that these two molecules may support apoptosis rather than paraptosis and autophagy processes in GB. This study is the first in the literature to investigate SB and CEL ‘s apoptotic and autophagic effects in GB.

When evaluating the effects of SB and CEL on cell cycles, it is reported in the literature that SB causes cell accumulation in G0/G1 phase [48] and blocks G1/S transition [34], while CEL induces G2/M arrest in various cancers, including glioblastoma [25, 36, 37]. In this study, it was observed that both SB and SB+CEL treatments led to apoptosis in the LN-405 cell line compared to the control, and significant differences were found between the groups. Furthermore, flow cytometry analysis demonstrated that SB triggered the apoptotic process in both cell lines. The presence of an increase in the SubG1 population in the CEL-treated group in the LN-405 cell line indicates the apoptotic effect of CEL. However, the discrepancy between the observed increase in the SubG1 population and the gene expression results suggesting apoptotic tendency in T98G cells may be attributed to the paraptosis process mentioned above. This also suggests that CEL may exert cell-specific effects at different levels.

## Conclusions

Our study has provided significant findings regarding the apoptotic, autophagic, and DNA repair mechanism effects of SB and CEL, in addition to new cytotoxicity data. Furthermore, it represents the first study in the literature investigating the impact of the combined use of SB and CEL on DNA repair pathways, cell cycle, autophagy, and apoptosis in GB. However, it is essential to acknowledge the limitations of our study. Nevertheless, both agents can penetrate the blood–brain barrier (BBB), which is an essential advantage for further in vivo studies. Further in vivo studies and patient-derived organoid studies should be conducted to bring us one step closer to GB treatment. The data obtained within the scope of this study have provided some key insights into GB chemotherapeutic resistance with the use of SB and CEL alone or in combination. In this context, it is believed to offer preclinical foundational data for a potential new treatment approach in GB.

## Data Availability

We confirm that the data supporting the findings of this study are available within the article.
